# Transcriptome Analysis Reveals the Role of Partial Lymphatic Vessel Ligation in Rats with Cirrhotic Portal Hypertension

**DOI:** 10.3390/cells15212000

**Published:** 2026-11-02

**Authors:** Zhenghao Wu, Guqing Luo, Jinbo Zhao, Qingxun Zhou, Hongjie Li, Chihao Zhang, Guangbo Wu, Qiang Fan, Xiaoliang Qi, Haizhong Huo, Jiayun Lin, Meng Luo, Lei Zheng

**Affiliations:** Department of General Surgery, Shanghai Ninth People’s Hospital, Shanghai Jiao Tong University School of Medicine, Shanghai 200011, China

**Keywords:** portal hypertension, liver cirrhosis, liver lymphatic system, lymphatic drainage, lymphatic endothelial cell, hepatic inflammation, immune response

## Abstract

**Highlights:**

- Partial ligation of hepatic lymphatics could functionally block the lymphatic outflow of liver.
- Obstruction of hepatic lymphatic drainage significantly exacerbated cirrhotic portal hypertension.
- Inflammatory signaling is associated with lymphatic dysfunction in portal hypertension and lymphatic ligation.

**Abstract:**

The liver lymphatic system plays an essential role during the progression of cirrhotic portal hypertension (PHT), featuring significantly enhanced lymphangiogenesis. However, the role of liver lymphatic functional drainage is understudied in cirrhosis. Therefore, this study aimed to investigate the effects of partial lymphatic vessel ligation (PLVL) of the liver on PHT. The rat model was established by common bile duct ligation (BDL), when PLVL was achieved via ligation of the portal and celiac lymph node. The severity of cirrhosis and PHT were evaluated, as well as macrophage infiltration and lymphangiogenesis. Bulk RNA-seq of liver was used to investigate the underlying mechanisms. Datasets of single-cell and bulk RNA-seq were obtained from the GEO database. Human dermal lymphatic endothelial cells (hLECs) were used for in vitro validation. Ligation of lymphatic drainage caused significant deterioration of liver cirrhosis and PHT in BDL rats. Our bulk RNA-seq suggested significant enrichment of vascular remodeling and angiogenesis-related pathways, as well as lipid metabolism-related biological functions in BDL rats after PLVL. Further analysis combined with public datasets identified several endothelial cell-specific upregulated genes in BDL and PLVL rats. LEC-derived upregulated genes were also identified from bulk RNA-seq of LECs isolated from mice liver, which were validated by incubating hLECs in TNF-α, LPS, TGF-β1. In the current study, we provide the first evidence that liver lymphatic drainage blockade aggravates BDL-induced cirrhotic PHT. LEC-associated secretory proteins might play a potential role in PHT progression, when hepatic inflammation and immune response are considered to be the essential regulatory mechanisms.

## 1. Introduction

The lymphatic system plays an essential role in fluid homeostasis, immune regulation, and lipid metabolism [1,2]. The liver is recognized as the largest lymph-producing organ, contributing to over 25–to-50% of lymph flowing through the thoracic duct. During the past decade, growing evidence has suggested the importance of the hepatic lymphatic vascular system in various liver diseases including liver cirrhosis and portal hypertension (PHT). However, compared to those in the skin, cancer, and cardiovascular system, the lymphatic system in the liver is understudied, despite its pivotal role and remarkable changes during development of chronic liver diseases [3].

Portal hypertension (PHT) is a syndrome featuring elevated portal pressure (PP) especially occurring at the end stage of chronic liver disease (CLD) [4]. Elevated intrahepatic vascular resistance (IHVR) due to liver microcirculatory dysfunction in cirrhosis is the major contributor to the development of PHT [5]. Defenestrated liver sinusoidal endothelial cells (LSECs) exhibited a vascular-like phenotype, provoking increased stiffness of sinusoidal microcirculation. An increase in IHVR also affected extrahepatic hemodynamics, leading to hyperdynamic splanchnic circulation, which further increased portal blood flow, and formed a vicious cycle.

In recent years, enhanced lymphangiogenesis in cirrhotic PHT has been observed in clinical patients and various animal models, and was considered as a compensatory mechanism to drain metabolic wastes and inflammatory factors. VEGF-C/VEGFR-3 has been proved to be the central signaling pathway promoting lymphangiogenesis during embryonic developments and disease progression [6,7]. Our previous studies have demonstrated that liver-targeted VEGF-C overexpression significantly activated hepatic lymphangiogenesis and alleviated liver cirrhosis and PHT [8]. Tanaka et al. reported that Schwann cell-derived VEGF-C promoted hepatic lymphangiogenesis, which was protective against liver fibrosis [3]. Jain et al. further highlighted the dynamic change in hepatic lymphangiogenesis during the development of cirrhotic PHT [9]. On the other hand, Juneja et al. focused on extra-hepatic lymphangiogenesis, when delivery of recombinant VEGF-C to the mesenteric lymphatic system ameliorated PHT, as well as complications including gut bacterial translocation and ascites [10]. Therefore, based on previous knowledge, VEGF-C/VEGFR-3 emerged as the major target of both intra- and extra-hepatic lymphangiogenesis to improve cirrhotic PHT and related complications. However, how lymphangiogenesis mitigates liver cirrhosis and PHT remains unclear. Few studies have focused on the role of altered lymphatic drainage, the key function of the lymphatic system in cirrhotic PHT.

Experimental obstruction of lymphatic drainage has been established in various models to study the effects of functional impairment on lymphatic system, including tail lymphatic ligation for tail lymphedema and deep cervical lymph-node ligation for meningeal lymphatic obstruction [11,12]. However, the utilization of liver lymphatic drainage obstruction is limited, so far. Intra-hepatic lymphatic vessels were thought to be located in three regions: the portal, hepatic venous, and sub-capsular areas. Lymphatic vessels located in portal tracts were admitted as the primary drainage site, covering approximately 80% of the lymph in liver [1]. The studies on the hepatic lymphatic system also mainly focused on these lymphatic capillaries, which collect lymph and eventually drain into portal and celiac lymph nodes [13,14]. With the established knowledge of drainage pathways, a recent preprint reported that removal of portal and celiac lymph nodes, followed by administration of gel, was able to block the liver lymphatic drainage [9]. However, it was hard to comprehensively evaluate the alterations of liver lymphatic drainage without preserving the target lymph nodes after establishing animal models. Another study highlighted the fact that the direct ligation of portal and celiac lymph nodes would also obstruct the hepatic lymphatic outflow, while similar surgical procedures have never been applied on PHT animals to investigate the effects of liver lymphatic drainage blockade [15].

Therefore, in the current study, we established the rat model of partial hepatic lymphatic vessel ligation (PLVL) on the basis of a bile duct ligation-induced rat model of cirrhotic PHT. We aimed to investigate the effects of lymphatic drainage obstruction on liver cirrhosis and PHT, trying to figure out the underlying mechanisms of liver lymphatic drainage during disease progression which might provide evidence for novel therapeutic targets and strategies.

## 2. Methods

### 2.1. Animals

Sprague Dawley (SD) rats (200–250 g, 6- to 8-weeks old) were procured from the Experimental Animal Center of the School of Medicine, Shanghai Jiao Tong University (Shanghai, China). All SD rats were maintained at our facility under specific pathogen-free conditions, including a temperature of 22 °C, humidity range of 40–60%, and a 12 h light/dark cycle with ad libitum access to food and water. A total of 51 rats were randomly assigned to the indicated experimental groups before the initiation of experiments. Firstly, the rats were randomly divided into two groups receiving common bile duct ligation (BDL) or sham operation (Sham). Differently operated rats were then randomly divided into the sham ligation group and PLVL group, respectively. Finally four groups of rats were involved in the current study: (a) Sham group (n = 7), (b) Sham + PLVL group (n = 10), (c) BDL group (n = 18), and (d) BDL + PLVL group (n = 16). BDL and PLVL were performed as previously described [15,16]. Briefly, for PLVL, we firstly identified portal lymph nodes beneath the portal vein, and celiac lymph nodes near the esophagus and stomach. Then, the efferent lymphatic vessels connected to the lymph nodes were isolated and ligated with 4–0 surgical sutures (Ethicon, Inc., Somerville, NJ, USA) (Figure 1A,B). Investigators were blinded to group allocation during outcome assessment and quantitative data analysis, whenever feasible. Ethical approval for all animal-related protocols was obtained from the Ethical Committee of the Shanghai Ninth People’s Hospital, Shanghai Jiao Tong University School of Medicine.

### 2.2. Statistical Analysis

Statistical analysis was performed by IBM SPSS Statistics 23 (SPSS, Inc., Chicago, IL, USA). Continuous variables are presented as means ± SEM. A one-way ANOVA followed by post hoc Tukey’s test was used for sets of data. Survival curves were compared using the log-rank test. *p* < 0.05 was considered statistically significant.

A detailed description of materials and methods is shown in the Supplementary Materials.

## 3. Results

### 3.1. PLVL Exacerbates Portal Pressure and Impairs Liver Function in BDL-Induced PHT Model

Rats were randomly assigned to four groups: Sham (n = 7), PLVL (n = 10), BDL (n = 18) and BDL+PLVL (n = 16). On the 28th day post-surgery, experimental rats were sacrificed for the assessment of hemodynamic parameters and collection of liver and serum samples. The liver lymphatic drainage of Evans blue was shown by significantly reduced Evans blue contents in LNs (Supplementary Figure S1A,B). The survival rate differed significantly between the Sham and BDL groups (100% vs. 50%), whereas the difference between the BDL and BDL+PLVL (50% vs. 56.25%) groups did not reach significance (Figure 1C). BDL surgery significantly reduced rat body weight, whereas PLVL had no significant effect (Figure 1D). PLVL surgery potentiated portal hypertension in the BDL rats. Nevertheless, PLVL surgery by itself does not elevate portal pressure above normal levels (Figure 1D). As expected, the BDL group resulted in significant liver injury compared to the Sham group (Figure 1E). Among the markers, serum alanine transaminase (ALT) and serum aspartate aminotransferase (AST) levels were further increased by PLVL surgery, but serum total bilirubin (TBIL) was not. Overall, although PLVL did not significantly affect the survival rate of BDL rats, the key phenotypes of liver cirrhosis and PHT were significantly aggravated.

### 3.2. PLVL Aggravates Hepatic Fibrosis and Inflammation in BDL-Induced PHT Model

Liver fibrosis was evaluated through morphometric analysis of the fibrotic area on sections stained with H&E, Masson’s trichrome, and Sirius Red (Figure 2A). Based on quantitative results, PLVL surgery failed to induce liver fibrosis alone, but significantly exacerbated fibrosis in the BDL rats (Figure 2A). The expression levels of *Acta2* and *Col1a1* in the liver further confirmed this conclusion (Figure 2B). In terms of inflammation, BDL surgery significantly upregulated pro-inflammatory cytokines (*Il1b*, *Il6* and *Tnf*) while downregulating the anti-inflammatory *Il10*. This inflammatory profile was further pronounced by PLVL surgery, which demonstrated a significant aggravation, specifically in *Il6* and *Tnf* levels (Figure 2B). *Vegfc*, as the most important driver of intrahepatic lymphangiogenesis, has been found to be upregulated in various liver disease models (including BDL) [3,16]. Here, PLVL surgery showed a trend of further upregulation, but it was not significant (Figure 2B).

### 3.3. PLVL Promotes Intrahepatic Macrophage Infiltration, Ductular Reaction and Lymphangiogenesis in BDL-Induced PHT Model

Macrophages infiltration is closely associated with liver fibrosis and lymphangiogenesis [17]. Hepatic macrophage infiltration was quantified by measuring the area ratio of CD68-positive regions surrounding the central veins. The BDL group showed a marked increase in intrahepatic macrophage infiltration compared to the Sham group. The most pronounced infiltration was observed in the BDL+PLVL group (Figure 3A). Additionally, we quantified macrophage counts within the medullary sinuses of lymph nodes distal to the portal lymph nodes. Unlike in the liver, the BDL+PLVL group showed a decrease in macrophage numbers within the lymph nodes compared to the BDL group (Figure 3B). This suggests that PLVL may exacerbate intrahepatic macrophage infiltration by reducing macrophage outflow. Finally, assessment of intrahepatic ductular reaction and lymphangiogenesis again demonstrated PLVL’s role in exacerbating BDL-induced liver pathology (Figure 3C).

### 3.4. Transcriptome Analysis Reveals the Underlying Mechanisms of PLVL Exacerbation of BDL-Induced PHT

Transcriptomic profiling of whole-liver samples across the four groups enabled us to investigate the mechanisms underlying the aggravating effect of PLVL on liver injury. Our initial analysis focused on the respective comparisons of BDL vs. Sham and BDL+PLVL vs. BDL, to identify upregulated DEGs. Applying a significance threshold of *p*-value < 0.05 and |log2 Fold Change| > 1.0, we detected 1967 and 1069 upregulated DEGs in the two comparisons, respectively. Among these, 490 DEGs were shared. We consider these genes to be key to how PLVL exacerbates BDL-induced liver pathology. Additionally, screening with the SignalP database identified 99 genes encoding putative secreted proteins (Figure 4A). The core DEGs and the secreted protein genes among them were subjected to KEGG pathway enrichment analysis with a significance threshold of *p*-value < 0.05. Following the filtration of liver-unrelated disease pathways, the top 20 most significant KEGG pathways were selected to generate scatter plots. As shown in Figure 4B, the core DEGs were notably enriched in pathways related to fibrosis, inflammation and angiogenesis. The activation of ECM–receptor interaction, focal adhesion and the TGF-beta signaling pathway is aligned with the worsening of hepatic fibrosis observed in the rats. In addition, leukocyte transendothelial migration, neutrophil extracellular trap formation and NF-kappa B signaling pathway are linked to heightened intrahepatic inflammation. In addition to angiogenesis, we also detected activity in the renin–angiotensin system and vascular smooth- muscle contraction related to vasoconstriction (Figure 4B). Furthermore, GSEA was performed to identify pathways represented by the genes with subtle expression changes. In the BDL vs. Sham comparison, we observed upregulation of classical pathways involved in fibrosis, inflammation, and angiogenesis, along with the semaphoring–plexin signaling pathway, which has been less studied in cirrhosis. More unexpectedly, the comparison between the BDL+PLVL and BDL groups revealed coordinated downregulation of multiple lipid metabolic pathways. This finding may suggest a potential link between lymphatic vessels and hepatic metabolic function (Figure 4C).

### 3.5. Identification of Endothelial-Associated Transcriptional Changes in Cirrhotic Liver Following PLVL

The direct connection between hepatic ECs and portal pressure underscores the value of examining liver-specific endothelial changes. To this end, we integrated single-cell RNA sequencing data from clinical healthy and cirrhotic liver samples (GSE136103) to identify genes enriched in endothelial cell populations that could be used to contextualize transcriptional changes observed in our liver tissue samples. The application of t-SNE dimensionality reduction to non-parenchymal cells (NPCs) identified 21 heterogeneous clusters, including ECs, B cells, NK cells, macrophages, hepatic stellate cells, and cholangiocytes, among others (Figure 5A, Supplementary Figure S2). *PECAM1* was employed as a universal endothelial marker, and subsequent stratification based on the relative expression of *PDPN*, *OIT3*, *ACKR1*, and *HEY1* across four cellular clusters delineated lymphatic ECs, liver sinusoidal ECs, vein ECs and artery ECs (Figure 5B). Cross-referencing the 185 endothelial-enriched genes (from cluster-based differential expression) with the 490 core DEGs yielded 17 overlapping genes (Figure 5C). These overlapping genes were subsequently evaluated in our whole-liver transcriptomic datasets, to determine whether their expression was altered in association with BDL and PLVL. As demonstrated in Figure 5D,E, these genes were significantly upregulated in both BDL vs. Sham and BDL+PLVL vs. BDL comparisons, while their expression in the public single-cell dataset was relatively enriched in endothelial populations. These findings identify a set of endothelial-associated candidate genes that may be relevant to the hepatic transcriptional response to BDL and PLVL. However, their cellular origin in our experimental liver samples cannot be directly established from the bulk RNA-seq data.

### 3.6. Implications of Lymphatic ECs in Liver Pathogenesis

Among liver ECs, lymphatic ECs remain relatively understudied, because of their low abundance and difficulties in specific identification [18,19]. Extending beyond the lymphatic vessel drainage function, we aimed to explore the direct involvement of lymphatic ECs in hepatic pathology. Integration of two murine LEC-enriched datasets (GSE255346 and GSE276093) with subsequent differential expression analysis revealed 182 and 393 upregulated genes in control–experimental (ischemia-reperfusion injury or BDL) group comparisons for each dataset. They showed an overlap of 13 genes with the previously identified endothelial-specific genes (Figure 6A). In whole-liver transcriptomic profiling of BDL vs. Sham and BDL+PLVL vs. BDL comparisons, *Mgp* and *CD63* exhibited significant upregulation in both comparisons, whereas *Mdk*, *Rbp7*, *Sema3a*, and *Fabp5* showed no significant changes in any comparison (Figure 6B). Aiming to elucidate LEC-mediated intercellular communication, we selected 7 putative secretory genes from the 13 overlapping candidates and employed STRING network analysis to pinpoint their potential downstream targets and specific receptors (Figure 6C). Regrettably, no specific receptors for MGP, FABP4, and SPARCL1 have been established. Furthermore, we systematically examined the expression patterns of these receptor genes across NPC populations and profiled their expression changes following BDL and PLVL procedures (Figure 6D,E). Single-cell mapping demonstrated pan-cellular presence of *Cd63* and *Tnfrsf1a*, compartmentalized expression of *Nrp1* and *Plxna2* in endothelial populations, and preferential enrichment of *Notch2* and *Ptprc* within immune cell clusters (Figure 6D). Unexpectedly, *CD63* demonstrated selective upregulation in response to both BDL and PLVL interventions, while other receptor genes maintained conserved expression levels (Figure 6E).

### 3.7. TNFα Upregulates Several Target Genes in Lymphatic ECs in Vitro

Due to limited research on intrahepatic lymphatic ECs, how the target genes identified in the above studies are regulated in lymphatic ECs remains unknown. As an initial investigation, we treated human lymphatic ECs in vitro with three common interventions and assessed the alterations in the mRNA expression of target genes (Figure 7A). TNF-α and LPS were respectively selected as classic endogenous and exogenous pro-inflammatory components in liver disease, and TGF-β1 as a classic pro-fibrotic component. Unexpectedly, the mRNA levels of several target genes were upregulated by TNFα intervention. LPS increased the expression of HSPG2, but TGF-β1 had essentially no effect on the regulation of these target genes (Figure 7B). These findings may suggest a close association between intrahepatic lymphatic endothelial cells and immunomodulation, as well as a central role for TNFα.

## 4. Discussion

In the current study, we have provided the first evidence that blocking liver lymphatic outflow on BDL rats significantly exacerbated liver fibrosis, inflammation, and portal pressure. Our further mechanistic investigations revealed that lipid metabolism, immunity, inflammation, extracellular matrix, and angiogenesis were tightly related to lymphatic obstruction in cirrhotic PHT, which was consistent with the biological functions of the lymphatic vascular system and drainage. In combination with scRNA-seq of human liver cirrhosis and bulk RNA-seq of isolated LECs of liver disease, we have identified several endothelial-derived factors, especially LEC-derived factors that potentially mediated PLVL-induced deterioration of cirrhotic PHT.

The differential response to PLVL between sham and BDL rats suggested that hepatic lymphatic obstruction might be better compensated under physiological conditions, but becomes consequential in cirrhotic PHT. BDL-induced disturbances in bile-acid metabolism and clearance may contribute to this impaired compensatory capacity, resulting in a more pronounced aggravation of liver cirrhosis and PHT after lymphatic obstruction [20].

Maintaining fluid homeostasis is one of the major functions of lymphatic vascular system. Hepatic lymph was generated when sinusoidal plasma filtered through fenestrated LSECs into the space of Disse between LSECs and hepatocytes. Then, the lymphatic capillaries located at portal areas would collect and drain the interstitial lymph to portal and celiac lymph nodes [1]. Our direct ligation of the outflow pathway could physiologically result in congestion of lymph within the lymphatic vessels. In the mice model of tail lymphedema, with a gross view of swelling tails, lymphangiogenesis and enlarged vascular lumen were observed in the cross-sections, even though the drainage function was impaired [11]. Although we did not find obvious swelling on the gross images of liver, our results showed similar histopathological alterations, indicating that lymphatic vessels at porta areas were significantly increased and dilated. Moreover, the production of lymph would be largely elevated under the condition of cirrhosis and PHT, accompanied with increased lymph flow in the thoracic duct, as previously reported [21]. Therefore, the increased lymph production and demand of sufficient drainage in BDL rats might account for the significant response and alterations upon PLVL, when sham rats might fully compensate the ligation with the limited lymph drained. Subsequently, PLVL-induced lymphatic congestion, especially in the cirrhotic PHT rats, impeded the removal of lymph in the space of Disse. The potentially increased hydrostatic pressure would then be further transferred to liver sinusoids, which directly resulted in increased IHVR and a deteriorated LSEC dysfunction.

As with the first dataset of bulk transcriptome describing the expression profile of BDL rats with lymphatic ligation, we also screened the genes encoding predicted secreted proteins. Based on these upregulated DEGs, fibrosis, inflammation, and immune-related pathways were enriched; as well as this, we noted the enrichment of angiogenesis, and vascular smooth-muscle cell contraction, which were widely recognized as core mechanisms during the development of cirrhotic PHT [4,22]. The enriched pathways also showed great consistency with our in vivo results, including alterations in portal pression, hepatic fibrosis, and inflammation. The GSEA also showed that TNF- and LPS-related functions were enriched, which is tightly related to our investigations on intrahepatic inflammatory cytokines, including TNF-α.

Regarding the significant enrichment of vascular angiogenesis and contraction, we made further insights into scRNA-seq of liver cirrhosis, mainly focusing on ECs. We identified 17 EC-specific genes in 490 upregulated DEGs between BDL vs. Sham and BDL+PLVL vs. BDL, which might play important roles in deteriorating liver cirrhosis and PHT. CD63 was reported as a marker of platelet activation in patients with portal vein thrombosis (PVT). CD41^+^CD63^+^ platelets were significantly increased in PHT patients with PVT, which was positively correlated with PHT-related complications [23]. The upregulation of *Lgals3*, which encoded galectin-3, also potentially drove the progression of PHT. A multi-center clinical trial demonstrated that galectin-3 inhibitor belapectin did not affect fibrosis, but was associated with HVPG reduction in MASH patients [24]. Aebp1 was considered as a key modulator of organ fibrogenesis in the liver and heart, the upregulation of which may aggravate PHT by increased liver fibrosis in BDL rats [25,26]. Moreover, *Lgals3*, *Tagln2*, *Aebp1*, *Itgb1*, and *Sox4* were reported to promote angiogenesis in various tumors [27,28,29,30,31]. The pro-angiogenic effects of these upregulated EC-derived genes might mediate the intra-hepatic angiogenesis and elevated IHVR in BDL rats.

The effects of PLVL suggested the effects of direct impairment of lymphatic drainage in cirrhotic portal hypertension. Therefore, the transcriptome alterations, specifically in LECs, were of great importance. LEC-derived factors might further bind to receptors and cross-talk with other liver cells, eventually driving disease progression. It is worth noting that the semaphoring–plexin signaling pathway and semaphoring–receptor binding were enriched by GSEA between BDL and sham livers, when a member of semaphorin family, Sema3a, was significantly upregulated in LECs from I/R and BDL mice. Sema3a was demonstrated to directly induce defenestration of LSECs, which was the key pathophysiological hallmark of IHVR increase in PHT [5,32]. Therefore, we speculated that LEC secretion of Sema3a played an essential role in defenestration and vascularization of LSECs, by interaction with NRP1 during the development of PHT. On the other hand, Fabp4, as the chaperone of fatty acid transportation, was one of the key regulators of lipid metabolism. Interestingly, the GSEA between BDL+PLVL and BDL showed significant enrichment of a group of lipid metabolism-related biological functions. It was evidenced that adipocyte FABP promoted liver fibrosis via cross-talk between LSECs and HSCs in BDL mice, which were major cells responsible for IHVR and fibrogenesis, respectively [33,34]. The upregulation of Fabp4 in LEC might be involved in similar mechanisms in BDL rats, as well. Moreover, as the detailed mechanisms of lipid metabolism were well-addressed in MAFLD/MASH, those roles in liver cirrhosis and PHT are understudied [35]. The engagement of the liver lymphatic system in lipid metabolism in the context of cirrhotic PHT warranted further investigation.

In our in vitro validation by hLECs incubated with different stimuli, it was the endogenous pro-inflammatory factor TNF-α, rather than exogeneous pro-inflammatory factor LPS or fibrogenic factor TGF-β1, that significantly induced upregulation of those secretory proteins identified by high-throughput data. The lymphatic system was widely acknowledged for its involvement in immune surveillance, especially in cross-talk and the transportation of immune cells with the help of various cytokines and chemokines [36]. During acute inflammation, TNF-α was proven to orchestrate neutrophil trafficking into lymphatic vessels [37]. Under inflammation in chronic liver disease, TNF-α was released by cell-intrinsic inflammasomes, recruiting cells of the adaptive immune system and fueling the hepatic inflammation [38]. As a significant upregulation of TNF-α in BDL+PLVL rats compared to BDL rats was elucidated, we speculated that TNF-α might exert pivotal effects after lymphatic ligation, altering the secretory profiles of LECs that mediated the cross-talk between LECs and immune cells. However, the detailed molecular mechanisms remain unclear, and need further exploration.

There are still several limitations to the current study. First, the bulk transcriptomic of the whole liver will be largely affected by the parenchymal cell hepatocytes. The changes in LECs, which only occupied a very small proportion of liver cells, might have minimal effects on the final results of bulk transcriptomics. Meanwhile, the direct functional and causal roles of LEC-associated factors were not investigated in the present study. Therefore, these factors should be considered candidate mediators, rather than established drivers of portal hypertension. Further studies using gain- or loss-of-function approaches will be required to determine their specific contributions and underlying mechanisms. Second, the changes in transcriptomics were not always consistent with changes at the protein level, especially for those secretory proteins. High-throughput sequencing at the protein level might help further clarify the mechanistic alterations after PLVL. Third, the current study lacks corresponding clinical data. It is hard to find a scenario in which hepatic lymphatic drainage is obstructed in cirrhotic patients. PLVL is currently a method of functional obstruction with preserved lymph node in situ. Further study might focus on the alterations of LECs in the lymphatic system, as suggested by transcriptomic analysis. Fourth, although our data revealed an association between increased TNF-α signaling and altered LEC gene expression, future studies using TNF-α blockade or genetic approaches targeting TNF signaling will be needed to further determine its contribution to the observed lymphatic endothelial alterations. Fifth, BDL is a cholestatic model, and does not fully recapitulate the etiologies of human cirrhotic PHT, which commonly include alcohol-associated liver disease, viral hepatitis, and MASH. Therefore, whether the effects of impaired hepatic lymphatic drainage aggravated other etiologies of cirrhotic PHT requires further investigations. Sixth, as the portal and celiac lymph nodes may also participate in lymphatic drainage from adjacent gastrointestinal and pancreatic tissues, PLVL cannot be considered completely liver-specific, and potential extrahepatic effects should, therefore, be taken into consideration.

## 5. Conclusions

In the current study, we found that impaired hepatic lymphatic drainage aggravated liver injury, fibrosis, and portal hypertension in BDL-induced cirrhotic rats. Impaired lymphatic drainage was accompanied by enhanced inflammatory and pro-fibrogenic responses, together with alterations in hepatic lymphatic endothelial cells. Transcriptomic analyses further identified changes in LEC-associated signaling and secretory factors that may contribute to the progression of portal hypertension. These findings provide evidence for an association between hepatic lymphatic dysfunction and cirrhotic portal hypertension, and warrant further investigation into the underlying mechanisms and potential therapeutic implications.

## Figures and Tables

**Figure 1:**
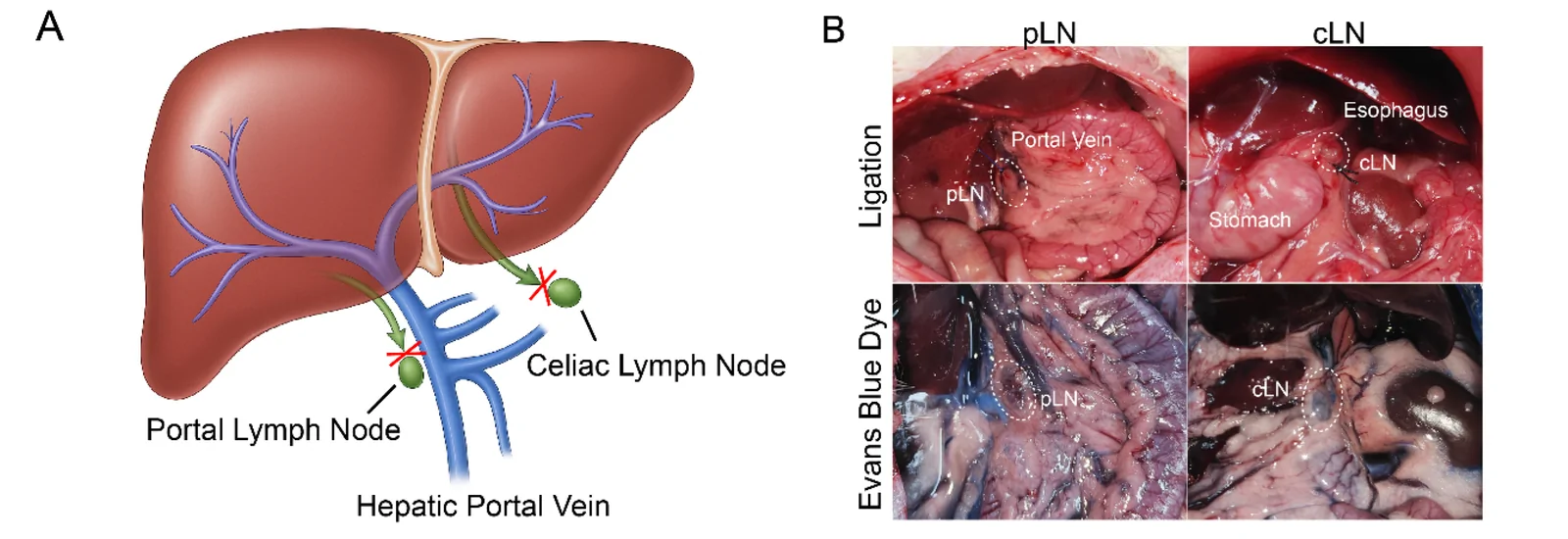

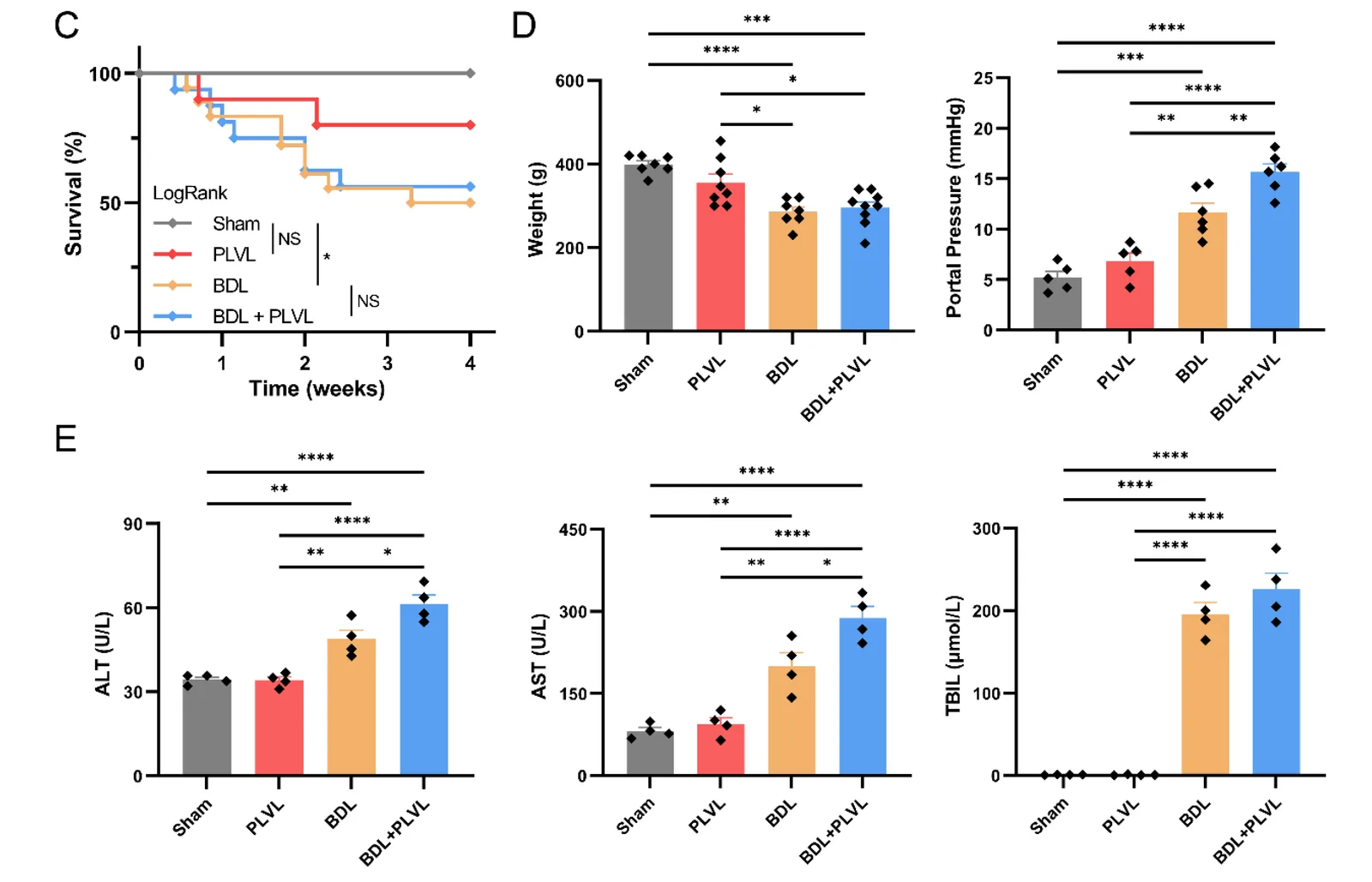
**PLVL aggravates portal pressure and liver dysfunction in BDL-induced PHT rats.** (**A**) Schematic diagram of partial lymphatic vessel ligation. Red X represents the blockade of lymphatic drainage. (**B**) Gross image of lymphatic vessel ligation and localization of portal and celiac lymph nodes by Evans blue injection via hepatic parenchyma. (**C**) Survival curve for each group after surgery, for 28 days (*p* > 0.05 for BDL group vs. Sham group). For Sham group, n = 7, for PLVL group, n = 10, for BDL group, n = 18, for BDL+PLVL group, n = 16. (**D**) General conditions and hemodynamic status of rats in each group. (**E**) Levels of blood serum ALT, AST and TBIL of rats in each group. Data represent the means ± S.E.M. Each diamond represents one biological replicate. * *p* < 0.05, ** *p* < 0.01, *** *p* < 0.001, **** *p* < 0.0001.

**Figure 2:**
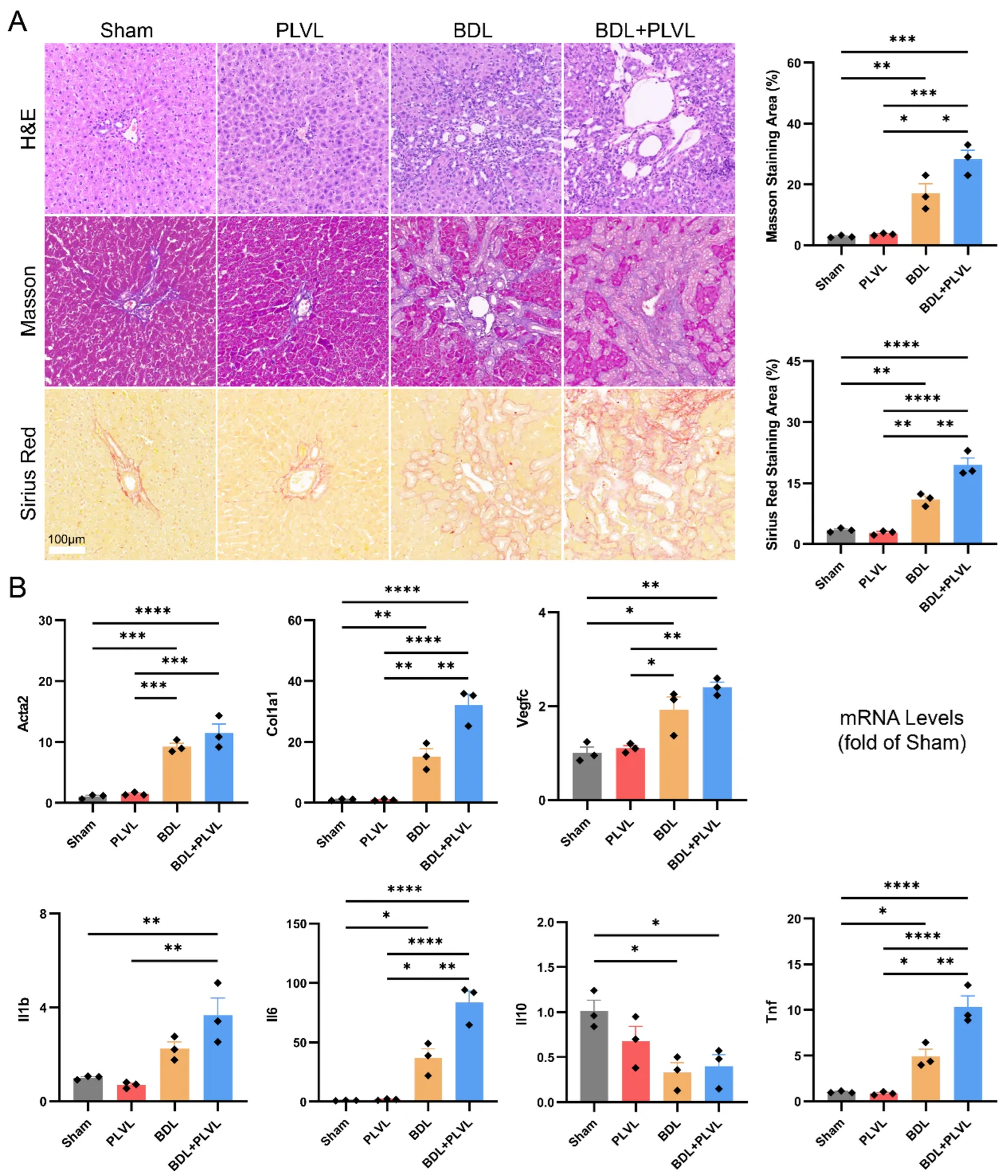
**PLVL exacerbates liver fibrosis and inflammation in BDL-induced PHT rats.** (**A**) H&E, Sirius Red, and Masson’s trichrome staining of liver sections with quantitative assessment of fibrotic areas in each group. (**B**) The mRNA expression of *Acta2*, *Col1a1*, *Vegfc*, *Il1b*, *Il6*, *Il10* and *Tnf* in liver tissues of each group. Data represent the means ± S.E.M. Each diamond represents one biological replicate. * *p* < 0.05, ** *p* < 0.01, *** *p* < 0.001, **** *p* < 0.0001.

**Figure 3:**
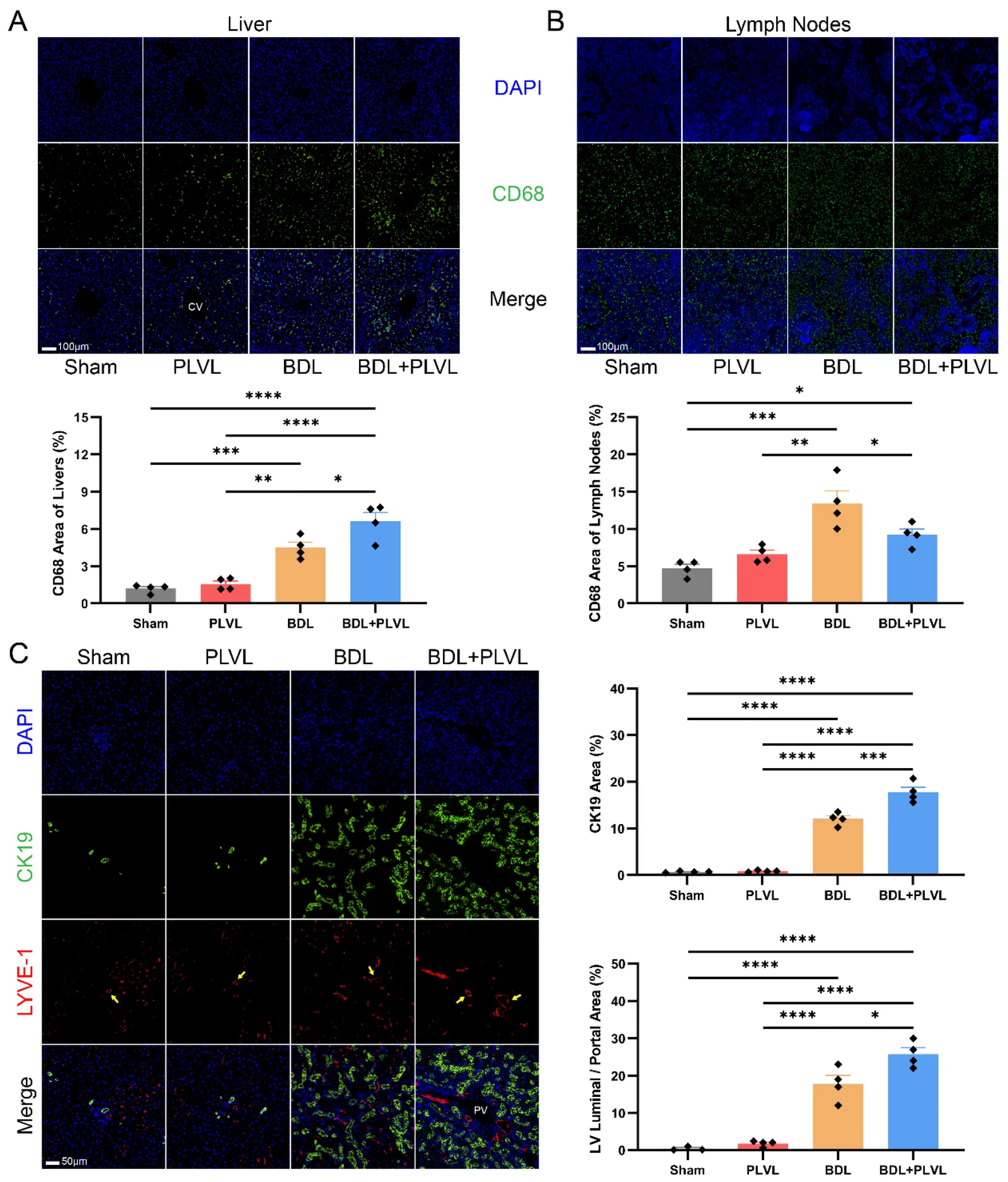
**PLVL enhances intrahepatic macrophage infiltration, ductular reaction, and lymphangiogenesis in BDL-induced PHT rats.** (**A**) Representative CD68 immunofluorescence of hepatic tissues and quantification of fluorescence-positive area in each group. CV, central vein. (**B**) Representative CD68 immunofluorescence of lymph nodes and quantification of fluorescence-positive area in each group. (**C**) Representative CK19 and LYVE-1 immunofluorescence of hepatic tissues and quantitative analysis of lymphangiogenesis and ductular reaction in each group. Yellow arrows showed lymphatic vessels around the portal tract. PV, portal vein. Data represent the means ± S.E.M. Each diamond represents one biological replicate. * *p* < 0.05, ** *p* < 0.01, *** *p* < 0.001, **** *p* < 0.0001.

**Figure 4:**
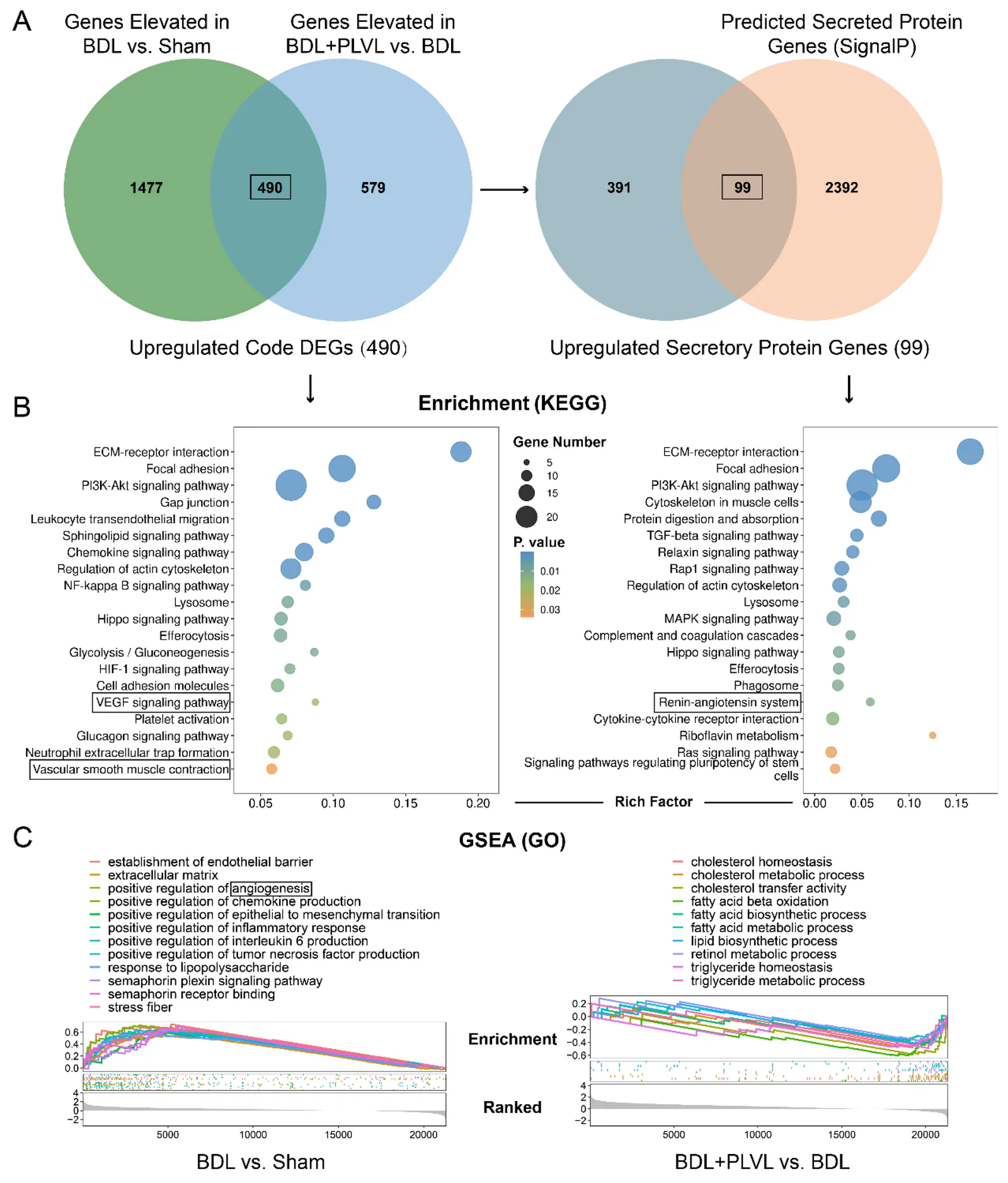
**Transcriptome analysis of PLVL-mediated effects in BDL-induced PHT rats.** (**A**) Co-expression analysis of up-regulated DEGs among different groups, followed by screening for secretory protein genes using SignalP. (**B**) KEGG pathway enrichment analysis of co-upregulated DEGs and their predicted secretory protein genes. (**C**) GSEA of the Sham vs. BDL and BDL vs. BDL+PLVL experimental groups.

**Figure 5:**
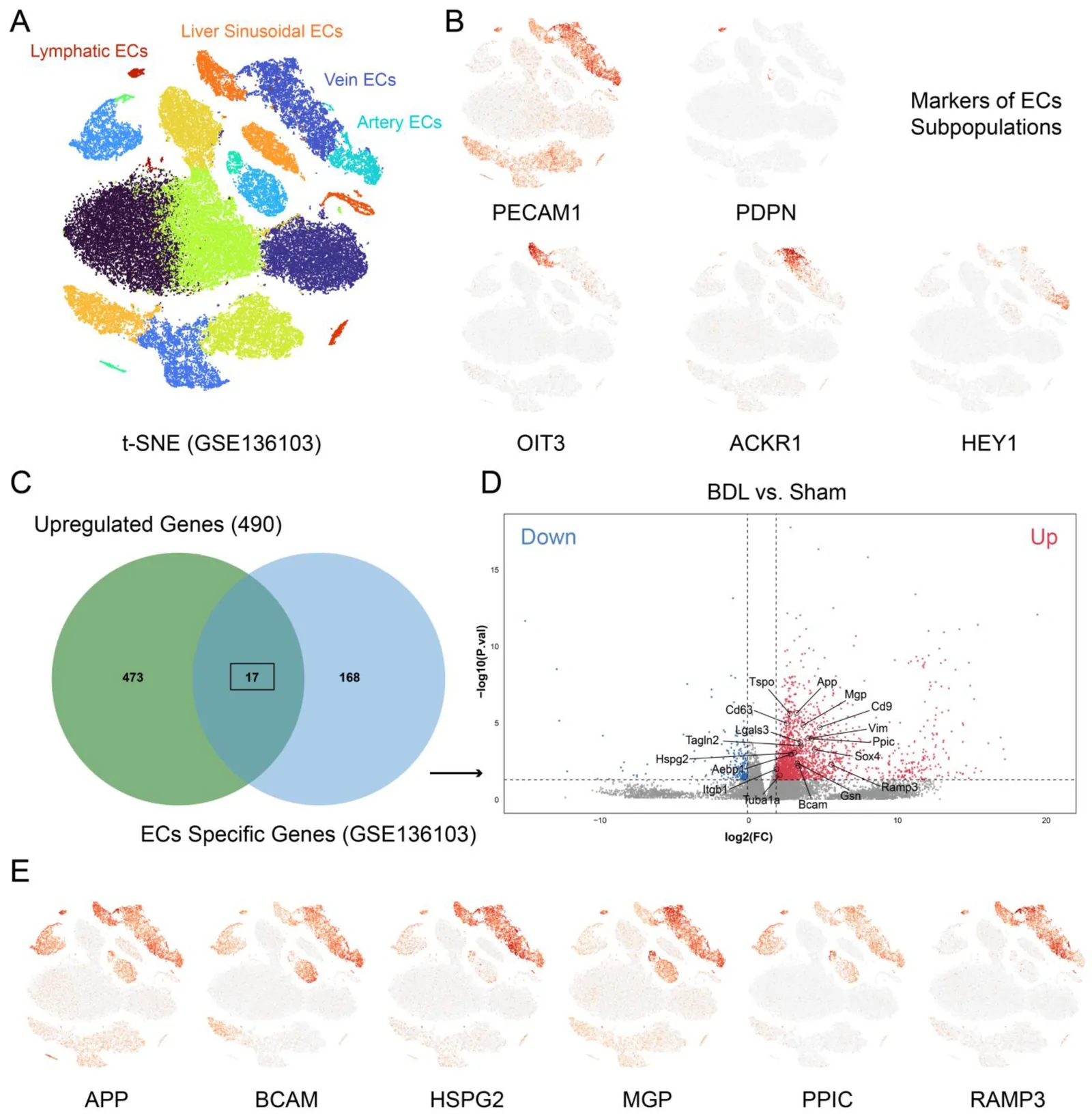
**Integration of clinical single-cell RNA sequencing data to investigate PLVL-mediated effects on hepatic endothelial cells.** (**A**) Visualization of clustering by t-SNE plot of integrated samples: five healthy and five cirrhotic human samples (from datasets GSE136103). (**B**) Cell type annotation of single-cell data using endothelial cell-specific markers. *PECAM1* (*CD31*) was used to define the overall ECs, *PDPN* for lymphatic ECs, *OIT3* for liver sinusoidal ECs, *ACKR1* for venous ECs, and *HEY1* for arterial ECs. (**C**) Co-expression analysis of co-upregulated DEGs with endothelial cell-specific genes from single-cell data. (**D**) Volcano plot of the endothelial-specific co-upregulated DEGs. (**E**) Expression distribution of selected DEGs in the single-cell data.

**Figure 6:**
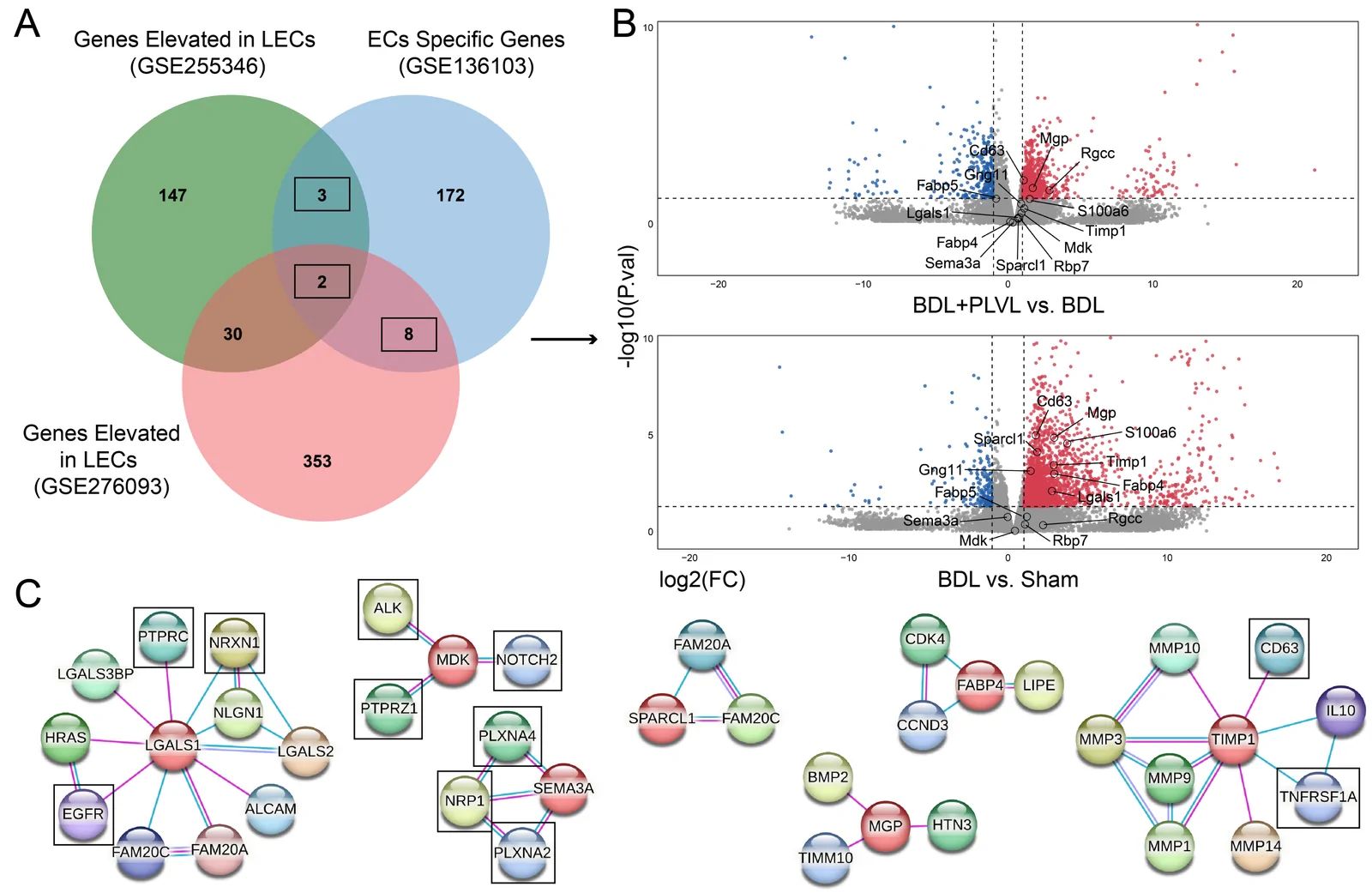

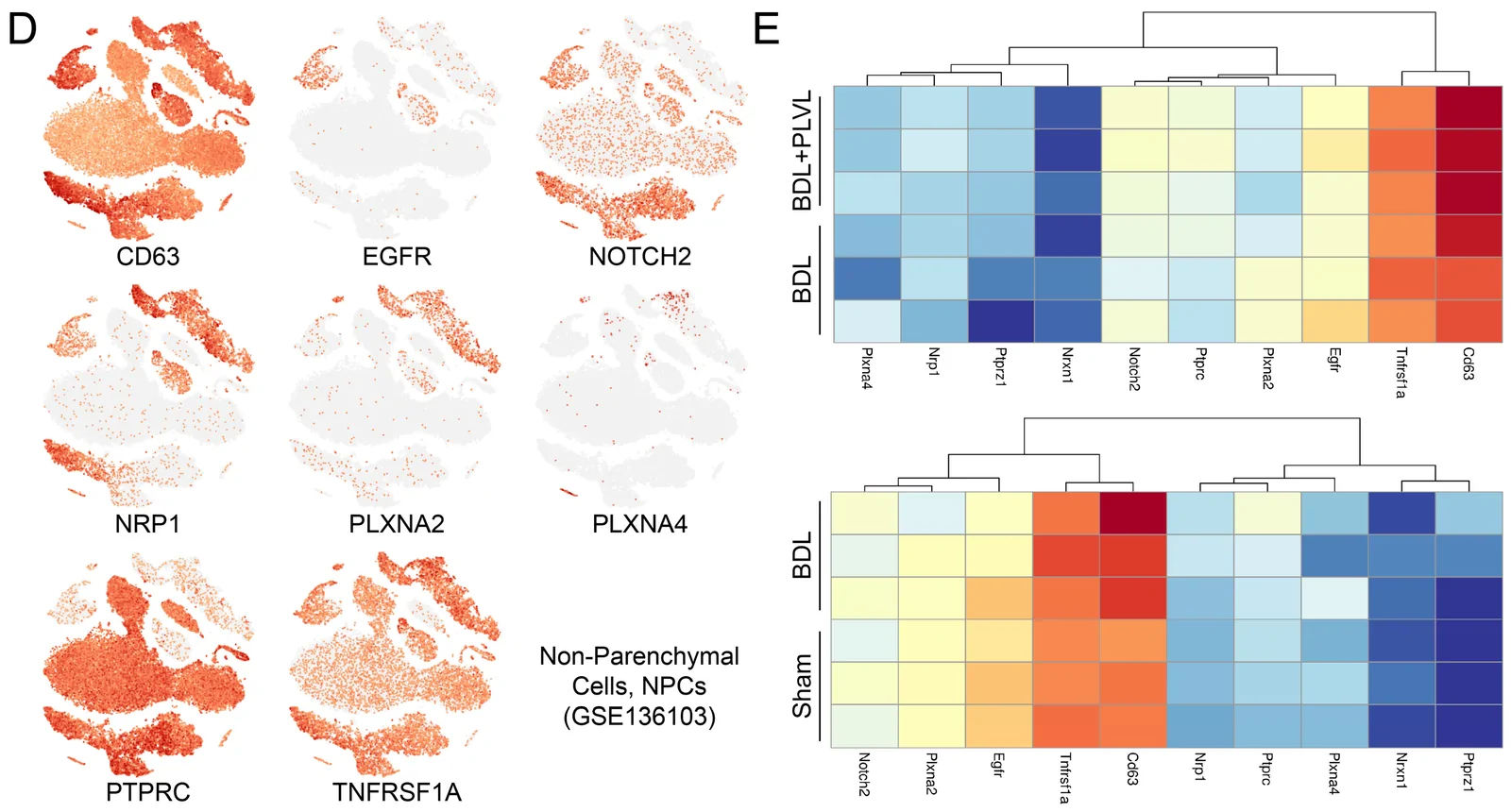
**Multi-transcriptomic analysis uncovers potential roles of lymphatic ECs in liver pathogenesis.** (**A**) Identification of up-regulated genes specific to lymphatic ECS in hepatic pathology (integrated analysis of datasets GSE136103, GSE255346 and GSE276093). (**B**) Differential expression of these genes in the whole liver across the BDL vs. Sham and BDL+PLVL vs. BDL experimental groups. (**C**) Detection of receptors for the secretory protein genes via STRING protein association networks. (**D**) Expression distribution of selected receptor genes in the single-cell data. (**E**) Hepatic expression levels of selected receptor genes across experimental groups.

**Figure 7:**
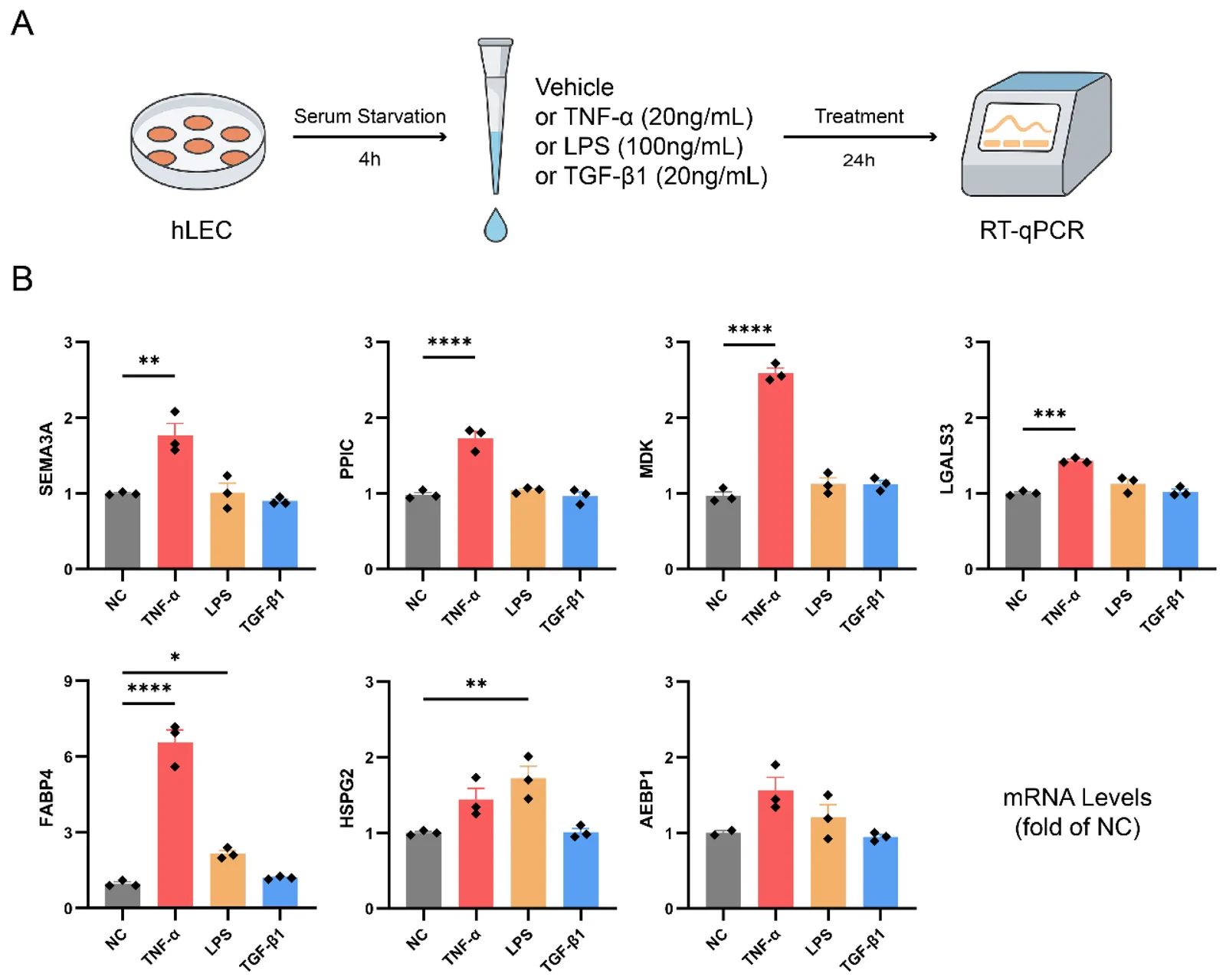
**Preliminary exploration of target-gene regulation in lymphatic ECs in vitro.** (**A**) Schematic diagram of the in vitro intervention in human lymphatic ECs. (**B**) The mRNA expression of *SEMA3A*, *PPIC*, *MDK*, *LGALS3*, *FABP4*, *HSPG2* and *AEBP1* in each group. Data represent the means ± S.E.M. Each diamond represents one biological replicate. * *p* < 0.05, ** *p* < 0.01, *** *p* < 0.001, **** *p* < 0.0001.

## Data Availability

The original contributions presented in this study are included in the article/Supplementary Materials. Further inquiries can be directed to the corresponding authors.

## Supplementary Materials

The following supporting information can be downloaded at: https://www.mdpi.com/article/10.3390/cells15212000/s1, Figure S1: Validation of blocked liver lymphatic drainage by evaluating Evans blue content in portal and celiac lymph node; Figure S2: Visualization of clustering by t-SNE plot of integrated five healthy and five cirrhotic human samples (from datasets GSE136103); Table S1: The primers used for reverse transcription and quantitative PCR reactions.
