# Supplementary material for: Transcriptome Analysis Reveals the Role of Partial Lymphatic Vessel Ligation in Rats with Cirrhotic Portal Hypertension
Source: Cells. 2026 Nov 2;15(21):2000. doi: 10.3390/cells15212000 (PMC13761516; doi:10.3390/cells15212000)

Supplementary Methods

Hemodynamic studies

The rats were anesthetized by 3% isoflurane. Briefly, PE50 tube was placed in the left femoral artery or portal vein of rats. After connecting the tube to the pressure sensor, portal vein pressure (PP) was measured by using an ALC-MPA multi-channel biological information analysis system (Shanghai Alcott Biotech Co., Ltd., Shanghai, China) (16). The zero-pressure point was defined at the level of right atrium.

Biochemical analysis

After hemodynamic measurements, serum was obtained from blood samples by centrifugation at 3000 rpm for 15 minutes at 4°C. The serum levels of alanine aminotransferase (ALT, S03030), aspartate aminotransferase (AST, S03040), and total bilirubin (TBIL, C120) were measured using commercial kits (Servicebio, Wuhan, China) following the manufacturer's instructions.

Liver lymphatic drainage evaluations

Evans blue (100 µl) was injected directly into liver parenchyma (right, left, and median lobes) and kept for 10 min under continuous anesthesia to allow the Evans blue migrating through the LVs and reach liver draining LNs. Evans blue dye was extracted from LNs in a 1.5 ml tube containing 300 µl of N, N-dimethyl formamide which was homogenized and incubated overnight at 55°C. Then the Evans blue content was measured with an excitation at 620 nm and an emission of 680 nm, and determined using a standard curve generated by 10, 5, 2.5, 1.25, 0.625, 0.3125, and 0 µg/mL of Evan blue.

Histological, immunohistochemical (IHC), and immunofluorescence (IF) examinations

The liver tissue from the right lobe was fixed by 10% formalin buffer (pH 7.4) and embedded in paraffin. For histological analysis, serial liver sections were subjected to hematoxylin-eosin (H&E), Masson, and Sirius Red staining, followed by random examination under light microscope by an experienced pathologist. The collagen deposition volume was calculated by an image analyzer (Image-Pro Plus, Media Cybernetics). The results were presented as the proportions of the stained areas.

For IF staining, serial liver and mesenteric tissue sections were incubated in primary antibodies against CK-19 (1:100, #12434, CST, USA), cluster of differentiation 68 (CD68, 1:100, #97778, CST, USA), and lymphatic vessel endothelial hyaluronan receptor 1 (LYVE1, 1:100, AF7939, R&D, USA) at 4 ℃ overnight. The corresponding Alexa Fluor 488 or 594-conjugated secondary antibodies were used for detection and DAPI was added to visualized the nuclei. Fluorescent images were captured by laser‐scanning confocal microscope (Leica TCS SP8, Leica Microsystems). All the images were analyzed with ImageJ software.

RNA-sequencing

Total RNA was isolated from the liver tissue using RNAiso Plus (TaKaRa, Japan). RNA quality was examined by gel electrophoresis and a Nanodrop spectrophotometer (Thermo, USA). For RNA-sequencing, samples from 3 groups and 6 biological replicates per group were used. Briefly, TruSeq RNA sample preparation kit (Illumina, USA) was used to construct strand-specific libraries, followed by sequencing using the Illumina HiSeq X. The raw data was converted into

Fastq format. The number of transcripts was calculated by fragments per kilobase of transcript per million fragments mapped (FPKM). Further bioinformatic analysis was performed by R 3.4.0 and corresponding R packages.

Single-cell RNA-sequencing (scRNA-seq)

scRNA-seq data (GSE136103) was obtained from GEO database and processed using the Seurat R package. Raw feature–barcode matrices generated by the 10x Genomics platform were imported and merged into a single Seurat object for downstream analysis. Quality control was performed by filtering cells based on the number of detected genes, total UMI counts, and the proportion of mitochondrial and hemoglobin gene expression.

Data were normalized using log-normalization. Highly variable genes were identified using the variance-stabilizing transformation (VST) method. The data were subsequently scaled and subjected to principal component analysis (PCA). Batch effects among samples were corrected using the Harmony algorithm. Cell clustering was performed using a shared nearest-neighbor graph-based approach with multiple resolutions tested. t-distributed Stochastic Neighbor Embedding (t-SNE) were applied for two-dimensional visualization.

Cell culture and treatment

Human dermal lymphatic endothelial cells (hLECs) purchased from Cellverse (Shanghai, China) were cultured in Endothelial Cell Medium (ECM, Sciencell, USA) supplemented with 5% fetal bovine serum (FBS), 1% endothelial cell growth supplement (ECGS), and 1% penicillin (100 IU/mL)/streptomycin (100 μg/mL) solution (P/S). The culture was maintained in a humidified incubator at 37°C with 5% CO_2. hLECs were incubated with TNF-α (20 ng/mL), LPS (100 ng/mL), or TGF-β1 (20 ng/mL) for 24 h for following experiments.

Quantitative real-time polymerase chain reaction (qRT-PCR)

The total RNA was extracted from tissues and hLECs by RNAiso Plus (TaKaRa, Japan). The cDNA was synthesized by RT SuperMix for qPCR (Vazyme, China). qRT-PCR was performed with ChamQ Universal SYBR qPCR Master Mix (Vazyme, China) in LightCycler® 480 Real-Time PCR System (Roche, Germany). The results from corresponding analysis software were presented as Ct values. The mRNA levels of targeted genes were normalized to GAPDH. The primer sequences used in this study were shown in Supplementary Table S1.

Supplementary Table S1

The primers used for reverse transcription and quantitative PCR reactions.

Human Gene	Forward primer sequence (5'-3')	Reverse primer sequence (5'-3')
SEMA3A	TCACCTGTTACCTCCAGTTTCC	AGACGCTGTAGGTCCCTTTG
PPIC	CTACCTCTCGTGCTTTGCGT	AAGAAGACCTTGGCCGTCAC
MDK	AAAGCAAAGGCCAAAGCCAAG	GACAAGGCAGGGCATGATTG
LGALS3	CAGCCAACGAGCGGAAAAT	TCCTTGAGGGTTTGGGTTTCC
FABP4	AAACTGGTGGTGGAATGCGT	GGTCAACGTCCCTTGGCTTA
HSPG2	CCTGACGGCCACTTCTACC	GCATTGTCACATTCACACCCTT
AEBP1	TATTACTTTGGGCCTCCTCCG	TGCTGTCCTCCTTTTTGGGT

Supplementary Figure S1

Validation of blocked liver lymphatic drainage by evaluating Evans blue content in portal and celiac lymph node.

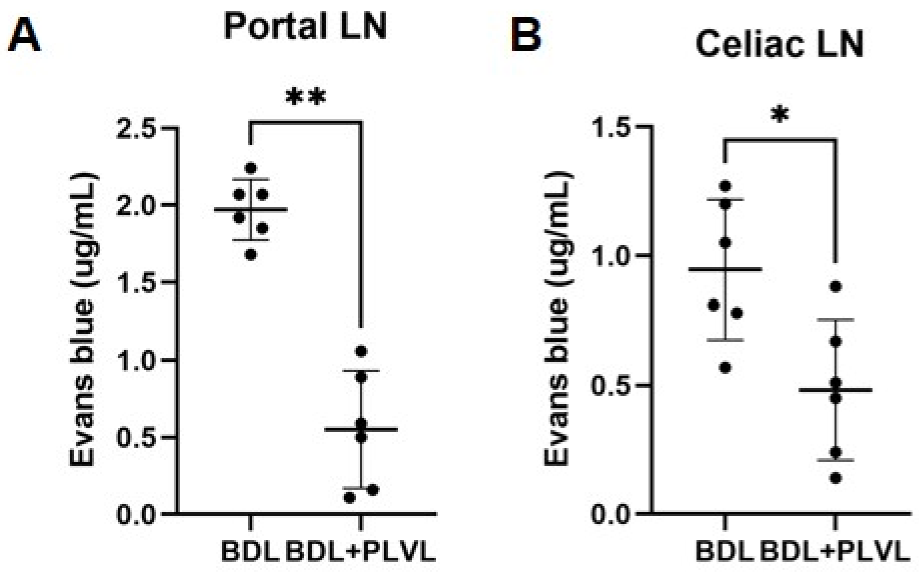

Supplementary Figure S2

Visualization of clustering by t-SNE plot of integrated five healthy and five cirrhotic human samples (from datasets GSE136103).

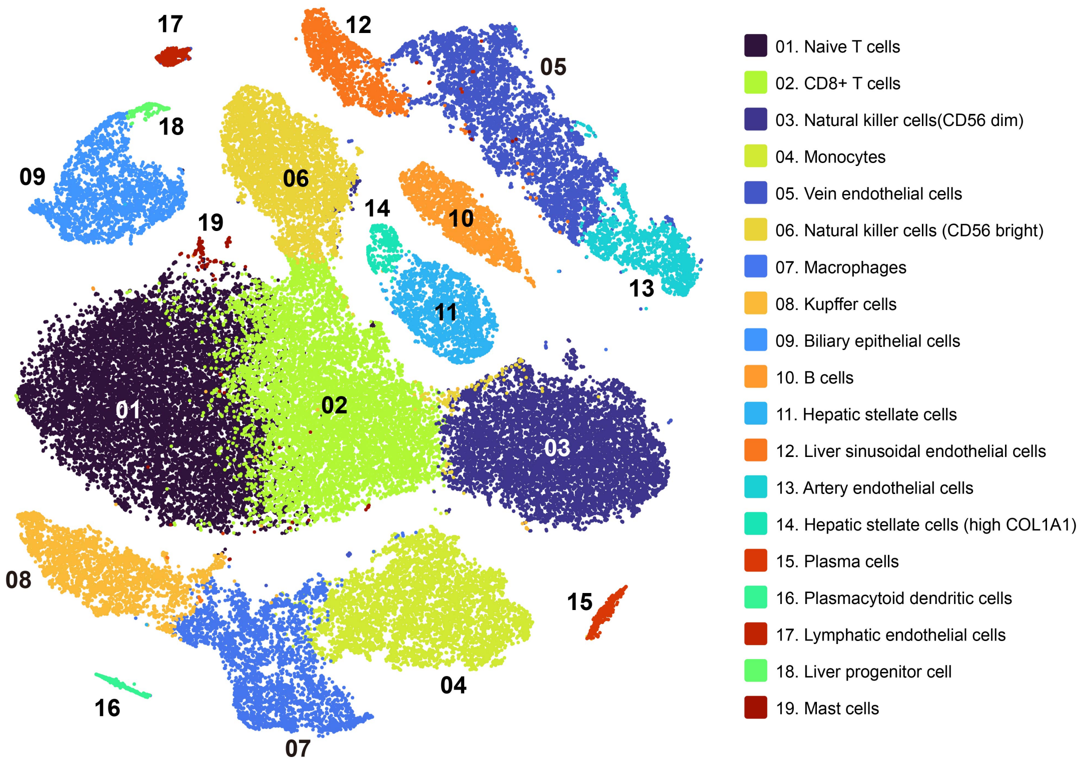

Supplement: Supplementary file 1 [file cells-15-02000-s001.zip › cells-4576464-supplementary.pdf]
